# Evaluating a Mobile Digital Therapeutic for Vasomotor and Behavioral Health Symptoms Among Women in Midlife: Randomized Controlled Trial

**DOI:** 10.2196/58204

**Published:** 2025-06-17

**Authors:** Jennifer Duffecy, Arfa Rehman, Scott Gorman, Yong Lin Huang, Heide Klumpp

**Affiliations:** 1 Department of Psychiatry, University of Illinois-Chicago, 912 S Wood, Chicago, IL, 60612, United States, 1 3124131225; 2 Chorus Health, San Francisco, CA, United States

**Keywords:** randomized controlled trial, digital therapeutic, women's health, behavioral health symptoms, behavioral, women, woman, physical symptoms, psychological symptoms, menopause, well-being, quality of life, digital technology, self-management, menopausal symptoms, efficacy, digital care, digital care program, app, vasomotor, depression, anxiety, sleep, mobile phone

## Abstract

**Background:** Perimenopausal and menopausal symptoms affect many women’s well-being and quality of life. Digital technologies, especially smartphones, allow self-management interventions for menopausal symptoms, but they are understudied.

**Objective:** We evaluated whether a novel digital care app, Caria, effectively reduced vasomotor and behavioral health symptoms of menopause.

**Methods:** We enrolled 149 women for a 6-week randomized controlled trial (app treatment: n=112; web-based educational control: n=37). Enrolled participants had problematic vasomotor symptoms and at least one elevated behavioral health symptom (depression, anxiety, or sleep issues). Web-based self-reported assessments (Hot Flush Rating Scale [HFRS], Patient Health Questionnaire Depression Scale-8 [PHQ-8], Generalized Anxiety Disorder-7, and Pittsburgh Sleep Quality Index [PSQI]) were conducted at baseline, 3 weeks, and 6 weeks.

**Results:** For hot flash severity (HFRS; treatment baseline mean 16.4, SD 6.7 to 6-wk mean 13.6, SD 6.6; control: baseline mean 19.1, SD 7.3 to 6-wk mean 17.8, SD 7.2), a repeated-measures ANOVA revealed main effects for time (*F*_2,262_=9.82; *P*<.001) and treatment arm (*F*_1,131_=6.08; *P*=.01) and a significant time × treatment arm interaction (*F*_2,262_=3.23; *P*=.04); the treatment arm showed lower hot flash severity than the control arm (*t*_147_=2.72; *P*=.007). For depression scores (PHQ-8; treatment baseline mean 14.0, SD 3.8 to 6-wk mean 11.2, SD 5.3; control baseline mean 15.0, SD 3.7 to 6-wk mean 13.4, SD 4.1), a repeated-measures ANOVA showed a main effect of time in the treatment arm (*F*_2,96_=15.2; *P*<.001) but not the control arm (*F*_2,40_=2.0; *P*=.15). Follow-up 2-tailed paired *t* tests in the treatment arm showed depression decreased from baseline to week 3 (*t*_49_=3.3; *P*=.002) and from weeks 3 to 6 (*t*_48_=2.3; *P*=.02). For sleep quality scores (PSQI; treatment baseline mean 10.7, SD 3.1 to 6-wk mean 10.0, SD 3.5; control baseline mean 11.5, SD 3.7 to 6-wk mean 11.0, SD 3.7), the repeated-measures ANOVA showed a main effect of time in the treatment arm (*F*_2,186_=7.8; *P*=.001) but not the control arm (*F*_2,62_=1.3; *P*=.28). Follow-up 2-tailed paired *t* tests in the treatment arm showed a significant decrease in sleep issues from baseline to week 3 (*t*_95_=3.9; *P*<.001) but no change from weeks 3 to 6 (*t*_93_=0.2; *P*=.81). Participants with elevated anxiety symptoms showed decreased symptoms in both the treatment and control groups. App engagement was high (average logins over 6 weeks: 53.2).

**Conclusions:** The findings highlight the potential of digital interventions for mitigating menopausal vasomotor and behavioral health symptoms. Significant improvements in the intervention group underscore the app’s effectiveness in providing relief from some of the most challenging aspects of menopause. This study contributes to the evidence supporting digital health interventions in managing menopausal symptoms, presenting a promising avenue for accessible and scalable solutions for women in midlife.

## Introduction

Menopause, defined as the permanent cessation of menstrual cycle, marks a major transition in a woman’s life [1]. It is typically preceded by perimenopause, a stage characterized by fluctuating hormone levels and the onset of various physiological changes [2]. These can include vasomotor symptoms (VMS) such as hot flashes (sudden, temporary onset of warmth in the body, flushing, and sweating) and night sweats (episodes of excessive sweating that happen during sleep), as well as depression, anxiety, and sleep disturbances [3]. In the United States, approximately 6000 women transition into menopause each day, many of whom face these challenges for years before and after menopause [4]. In recent years, research has begun to unveil the profound effects of these symptoms on women’s well-being, work performance, and careers, as well as the associated economic burden [5,6]. For example, sleep disturbances due to hot flashes, night sweats, and insomnia often result in daytime fatigue, impairing cognitive function, and productivity. Studies have found that menopausal symptoms can lead to women taking more sick leave, reducing working hours, changing roles, or even leaving the workforce altogether [7,8].

Given that menopause symptoms can persist for several years, the long-term impact of untreated symptoms on women’s quality of life and work performance is significant, resulting in substantial costs due to increased health care utilization and productivity losses [5,9]. The annual cost of menopausal symptoms in the United States alone is estimated to be US $26.6 billion, including US $1.8 billion due to productivity losses [10]. As the life expectancy of women continues to rise and midlife and older women make up one of the fastest-growing employment groups, it becomes increasingly important to address the considerable impact of menopausal symptoms through effective solutions [9,10].

Despite the impact of menopausal symptoms, there has been a notable lack of research on effective and accessible interventions. Common treatments for vasomotor and behavioral symptoms among women in midlife include pharmaceuticals such as menopausal hormone therapy and selective serotonin reuptake inhibitors [11-13]. However, menopausal hormone therapy is contraindicated for some women (such as survivors of breast cancer) [13], or some women may seek nonpharmacological interventions [12]. Behavioral interventions, such as cognitive behavioral therapy (CBT), have been recommended as a potential treatment for menopausal symptoms [14], with The Menopause Society rating the support for CBT to address menopause symptoms as level 1 (good and consistent evidence) [15]. CBT interventions focus on changing negative thought patterns and behaviors, promoting adaptive coping strategies, and enhancing emotional regulation [16,17]. Such interventions have been shown to improve psychological symptoms associated with health conditions such as insomnia, depression, cancer, chronic pain, and premenstrual syndrome [18-21], but would benefit from additional study in the context of menopause.

Recent studies have explored the efficacy of behavioral health interventions, such as CBT and mindfulness, which involve nonjudgmental awareness of internal state and surroundings [22], in improving health outcomes among menopausal women with promising results [23,24]. In a single-blind randomized controlled trial (RCT) by Greene et al [25], group-based, in-person CBT showed a reduction in VMS interference and bothersomeness, as well as depressive symptoms and sleep difficulties. In a 2018 study of a self-help unguided CBT booklet, results showed a reduction in problem rating of hot flashes and night sweats, in addition to improvements in sleep and work impairment [24].

Clinical guidelines recommend evidence-based psychotherapies such as CBT as the primary treatment for depressive symptoms during the menopause transition [25,26]. However, in-person or clinician-administered behavioral interventions can be costly and difficult to access for patients [27,28]. Digital health technology, such as smartphone apps, provides the opportunity to deliver effective behavioral treatments for individuals experiencing difficulties with menopause (eg, distress related to hot flashes, depression, and anxiety) in more scalable and cost-effective ways [29-31]. The increased convenience and privacy of digital approaches also have the potential to address health equity gaps by reducing barriers due to travel, logistics, and stigma. They also provide an additional treatment option for women who prefer alternatives to traditional treatments for menopausal symptoms, such as menopause hormone therapy, which is associated with an increased risk of certain health conditions [32].

In recent years, several studies have evaluated the efficacy of smartphone-based interventions for anxiety, depression, and insomnia (among others) [33-36]. However, there is little research on mobile-based behavioral interventions tailored for menopause. Meta-analyses have found few digital solutions for menopause, and most were focused on decision-support for menopausal treatments or content related to education and clinical guidelines, but few interventions for symptom relief [37,38]. Another 2019 study by Atema et al [39] investigated the efficacy of internet-based CBT and found significant reductions in the perceived impact of hot flashes and improved sleep quality. However, the sample was limited to survivors of breast cancer and only enabled the investigation of effects on treatment-induced menopausal symptoms. These studies highlight the dearth of mobile-enabled, self-guided, evidence-based behavioral health interventions for managing menopausal symptoms.

The objective of this study was to assess the efficacy of a novel mobile-based digital care program (DCP) called Caria (Chorus Health Inc) in reducing vasomotor and behavioral health symptoms (eg, hot flashes, depression, anxiety, and poor sleep quality) and explore treatment variables including length of treatment and treatment engagement. Based on evidence from prior research about the efficacy of behavioral interventions for treating menopausal symptoms, we hypothesized that the DCP would reduce VMS-related distress compared to the control group, as measured by the Hot Flush Problem Rating Scale (primary outcome). We also investigated the impact on depression, anxiety, and sleep quality (exploratory outcomes).

## Methods

### Study Design

The study was a 2-armed, randomized, controlled trial of participants with symptoms of menopause. Participants were recruited via digital channels, provided informed consent, and completed the intervention fully remotely. We followed CONSORT (Consolidated Standards of Reporting Trials) guidelines for reporting this trial.

### Ethical Considerations

The study was approved by the Institutional Review Board at the University of Illinois Chicago (STUDY2021-0282) and registered with clinicaltrials.gov (NCT04882982) on May 6, 2021. Participants completed an informed consent and had the ability to discontinue participation at any time. All participant data were deidentified prior to analysis. Participants were financially compensated with US $10 for the baseline, US $15 for the 3-week, and US $20 Amazon gift cards for the 6-week assessments. Those who were screened via MTurk received an additional US $1 for completion of the screening survey.

### Recruitment

People were recruited between February and October 2022 via advertisements placed on Facebook, Instagram, and Amazon Turk describing a “menopausal symptoms research study.” After clicking on the advertisement, potential participants were given additional study information, as well as the link to the screening questionnaire on Qualtrics, hosted by the University of Illinois at Chicago. The screening questionnaire assessed demographic characteristics and inclusion criteria for the study. People were eligible if they had (1) variable menstrual cycles (“If you have had a period in the last 12 mo, has it been irregular;” menopausal transition) or were more than 1 year from their last menstrual period (postmenopause); (2) hot flash or night sweats experienced as problematic (as indicated by an average score of two or higher on three items of the Hot Flush Rating Scale [HFRS]); (3) elevated score on at least one measure of distress—either depression (Patient Health Questionnaire Depression Scale-8 [PHQ-8] of 10 or greater), sleep (Pittsburgh Sleep Quality Index [PSQI] of 5 or greater), or anxiety (Generalized Anxiety Disorder-7 [GAD-7] of 10 or greater); (4) own an apple device able to run the Caria App; (5) English speaking; and (6) aged 30‐60 years. Menopause status was not fully characterized as the study was seeking to enroll a general population. The presence of other health conditions, such as polycystic ovary syndrome, was not assessed. Internet and mobile or computer literacy were implicit criteria, as participants must be proficient in all three to enroll and access the intervention. Participants were quasi-anonymous, as emails were collected and used as a means of communication. Technical measures (eg, disallowing the same device, IP, or location coordinates to enroll more than once) were used to prevent and detect the use of multiple identities.

A total of 149 people consented to participate in the study and were randomized to receive either the Caria DCP for 6 weeks (experimental group) or to receive a link to the publicly available The Menopause Society website with educational content about menopause symptom management (control group). Participants were followed up at the 3-week and 6-week mark.

### Allocation Strategy or Randomization

Participants were randomized into the trial via an algorithm that randomly seeded and shuffled participants into their groups using a treatment-to-control ratio of approximately 3:1, respectively. To prevent selection bias, the researchers had no way of knowing which participants would be allocated to the DCP or control group (allocation concealment). This randomization allocation was chosen as the aims of the trial included exploring the dimensions of treatment length, usability, and engagement. The utilization of unequal allocation is an appropriate method for evaluating clinical utility [40]. After participants were randomized, the intervention group received an email with written and video instructions on how to download and use the Caria app. Control participants received an email with The Menopause Society website link [41]. Neither the researchers nor the participants were blinded to the group allocation due to the design of the study.

### Procedures or Study Flow

Participants were first sent a link via email to fill out a baseline questionnaire before starting their respective interventions (DCP vs control). Those in the DCP arm were provided with written and video instructions on app installation and use. Participants were told that they had access to DCP for 6 weeks, but were not explicitly told how often or when to use it. Those in the control group were told that they had access to a website with resources on menopause for 6 weeks. Follow-up surveys were sent to both groups at the 3-week and 6-week mark. At the end of the study, all participants were debriefed via email, and both groups were provided with a link to the Caria app and The Menopause Society Website as resources.

### Measures

All measures were self-assessed via web-based questionnaires at 3 time points—baseline, 3 weeks, and 6 weeks. Participants also completed a screening assessment. The selected measures are all commonly administered digitally.

The HFRS is a 5-item self-reported assessment that measures the frequency of hot flushes (in days or weeks) and hot flush problem rating (sum of 3 items ranging from 1 to 10 each) [42]. The Hot Flush Problem Rating assesses the perceived distress associated with hot flushes and their impact on various aspects of psychological well-being, daily functioning, and social interactions. An average score of 2 or higher on 3 items of the HFRS is considered problematic. Prior studies using the HFRS have demonstrated reasonable test-retest reliability and concurrent validity [42,43]. Using Cronbach α, internal consistency among our sample was excellent (α=0.94).

The PHQ-8 is an 8-item self-reported questionnaire that measures current depression [44]. It is well validated as a diagnostic measure across clinical studies. Scores for each item range from 0 to 3, and total scores are calculated by summing the 8 items and can range from 0 to 24. Higher scores equate to higher depression. Scores of 10 or greater are considered clinically significant. The PHQ-8 is considered to be a reliable and valid measure for screening depression in the general population [45]. Internal consistency among our sample was good (Cronbach α=0.85).

The PSQI is a 19-item self-reported questionnaire that measures sleep quality [46]. A global PSQI score is calculated by summing 7 component scores (comprised of: subjective sleep quality, sleep latency, sleep duration, sleep efficiency, sleep disturbance, use of sleep medication, and daytime dysfunction). Scores of 5 and above indicate sleep problems. Reliability and validity of the PSQI are considered acceptable [47,48]. Internal consistency among our sample was acceptable (Cronbach α=0.60).

The GAD-7 is a brief 7-item self-reported measure of generalized anxiety [49]. Scores for each item range from 0 to 3, and total scores are calculated by summing the 7 items and can range from 0 to 27. Higher scores equate to higher levels of anxiety. Scores of 10 or greater are considered clinically significant. The GAD-7 has shown high reliability and validity for screening generalized anxiety in the general population [50]. Internal consistency among our sample was excellent (Cronbach α=0.92).

Adherence to the intervention was measured using the number of logins as the primary marker. Data on app persistence (defined in days, from account creation on the app to the end of 6 wk intervention) and the total time spent on the app in that period were also collected (Table 1).

The Usefulness, Satisfaction, and Ease Questionnaire (USE) is a widely accepted instrument for usability that measures satisfaction, usefulness, ease of use, and ease of learning using a 1‐7 Likert scale [51]. Higher numbers indicate greater usability and satisfaction. It has demonstrated validity for evaluating the subjective usability of software [52]. In this study, a brief version of the measure (5 items) covering Usefulness (1 item), Ease of Use (1 item), Ease of Learning (1 item), Satisfaction (2 items), and Total Scale was used to measure overall usability of and satisfaction with the intervention. Internal consistency of the USE among our sample was high (Cronbach α=0.96). USE ratings are listed in Table 2.

**Table 1.** Participant demographics*.*

Demographics	Overall	Intervention:Caria	Control:TMS^a^ website	Tests of independence(*P* value)
Age (years)				.48
Mean (SD)	47.72 (7.68)	47.78 (7.78)	48.46 (7.45)
Range	30-60	30-60	30-60
Menopause status, n (%)				.81
Perimenopause	86 (57.7)	48 (42.9)	15 (40.5)	
Menopause	63 (42.3)	64 (57.1)	22 (59.5)
Ethnicity, n (%)				.04
Hispanic	12 (8.1)	12 (10.7)	0 (0)	
Non-Hispanic	137 (91.9)	100 (89.3)	37 (100)
Race, n (%)				.51
American Indian/Alaskan Native	2 (1.3)	2 (1.8)	0 (0)	
Asian	7 (4.7)	6 (5.4)	1 (2.7)
Black or African American	13 (8.7)	8 (7.1)	5 (13.5)
More than one race	9 (6)	8 (7.1)	1 (2.7)
Native Hawaiian/Pacific Islander	0 (0)	0 (0)	0 (0)
White	118 (79.2)	88 (78.6)	30 (81.1)
Employment status, n (%)				.09
Full-time (>32 h/wk)	84 (56.4)	64 (57.1)	20 (54.1)
Part-time (<32 h/wk)	29 (19.5)	18 (16.1)	11 (29.7)
Not employed, seeking	4 (2.7)	4 (3.6)	0 (0)
Not employed, not seeking	17 (11.4)	16 (14.3)	1 (2.7)
Retired	5 (3.4)	2 (1.8)	3 (8.1)
Disabled	8 (5.4)	6 (5.4)	2 (5.4)
Student	1 (0.7)	1 (0.9)	0 (0)
Declined/Unknown	1 (0.7)	1 (0.9)	0 (0)
Education level, n (%)				.41
<12th grade	1 (.7)	1 (.9)	0 (0)	
High school graduate or GED^b^	12 (8.1)	11 (9.8)	1 (2.7)
Some college or associate’s (2 y) degree	47 (31.5)	32 (28.6)	15 (40.5)
Bachelor’s (4 y degree)	53 (35.6)	39 (34.8)	14 (37.8)
Graduate degree or more	36 (24.2)	29 (25.9)	7 (18.9)
Income level (in US $), n (%)				.81
<10,000	2 (1.3)	1 (.9)	1 (2.7)	
10,000-25,000	11 (7.4)	7 (6.3)	4 (10.8)
26,000-50,000	23 (15.4)	18 (16.1)	5 (13.5)
51,000-75,000	34 (22.8)	27 (24.1)	7 (18.9)
76,000-100,000	32 (21.5)	25 (22.3)	7 (18.9)
>100,000	46 (31.5)	34 (30.4)	13 (35.1)
Marital status, n (%)				.24
Single, never married	29 (19.5)	20 (17.9)	9 (24.3)	
Married	87 (58.4)	65 (58.0)	22 (59.5)
Separated	6 (4)	6 (5.4)	5 (13.5)
Divorced	23 (15.4)	18 (16.1)	1 (2.7)
Widowed	1 (.7)	0 (0)	1 (2.7)
Other	3 (2)	3 (2.7)	0 (0)

a TMS: The Menopause Society .

b GED: General Education Development.

**Table 2.** App engagement and usefulness ratings (Caria-group only; n=100).

Measure	Value (n=100)
Number of sessions	
Mean (SD)	53.24 (66.97)
Range	1‐398
Time on app (hours)	
Mean (SD)	3.77 (15.57)
Range	0.01‐157
App persistence (days)	
Mean (SD)	30 (15.73)
Range	0‐42
USE^a^—Usefulness of app	
Mean (SD)	4.96 (1.86)
Range	1-7
USE—Ease of use	
Mean (SD)	5.25 (2.06)
Range	1-7
USE—Ease of learning app	
Mean (SD)	5.31 (2.02)
Range	1-7
USE—Satisfaction with app	
Mean (SD)	5.04 (2.02)
Range	1-7
USE Total Score, mean (SD)	20.6 (7.5)

a USE: Usefulness, Satisfaction, and Ease Questionnaire.

### Interventions

#### Control Condition

Participants randomized to the control group received a link to educational material on menopause symptoms from The Menopause Society website, a well-respected menopause organization. The Menopause Society website contains hyperlinks to internal and external resources and averages less than one page of text per web page.

#### Active Condition—Caria DCP

Participants randomized to the active intervention group (ie, Caria DCP) received a link to download Caria (version 1.9.12), a smartphone-based app that includes a self-guided DCP designed for menopause symptoms. No additional cointerventions were provided. Participants were provided free access to the app (available on iOS) for the duration of the study, along with written and video instructions on app installation and use. There were no bug fixes or content changes between the study commencement and the study conclusion.

**Figure 1.**
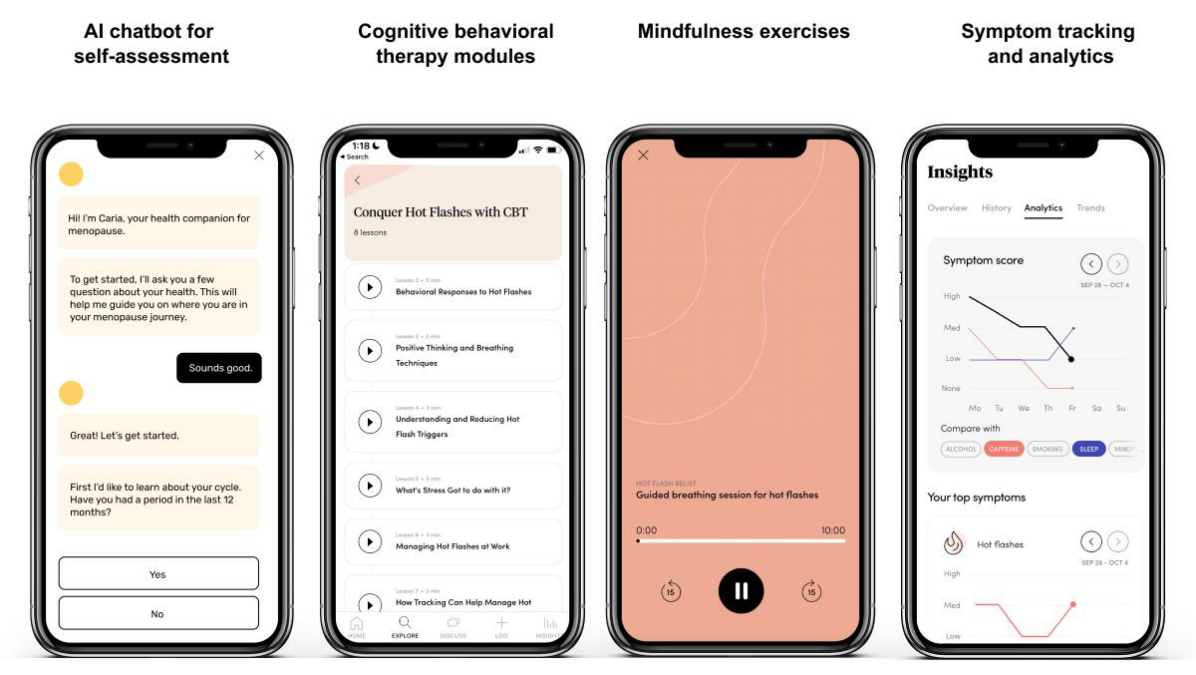
Examples of Caria app features and digital care program content.

The Caria app includes an artificial intelligence (AI)-chatbot-assisted menopause symptom assessment and a 6-week multimodal, self-guided DCP. For each day of the program, participants receive a number of modules and tasks to complete, including a CBT lesson, a mindfulness-based exercise in the form of a guided audio session, a mobility exercise video, and a community discussion topic where participants can receive or provide peer support asynchronously with other app users. Video content on the app is approximately 2‐10 minutes in duration, and the text content is approximately 800‐1000 words (roughly a 2‐5 min read). Figure 1 shows examples of app features. The program design was informed by prior research and care guidelines based on the efficacy of approaches such as CBT, mindfulness, and mobility for menopausal symptoms [25,27,53,54].

The DCP is divided into 6 weeks, covering themes such as understanding the menopausal transition (wk 1) and learning behavioral approaches to managing common symptoms, with a particular focus on anxiety and depression (wk 2), hot flashes and night sweats (wk 3), and sleep (wk 4). The DCP also covers behavioral techniques for improving mental wellness during the menopause transition (wk 5) and managing age-related health conditions and comorbidities, such as heart health and cognitive health (wk 6). All modules are available to users at any time and can be accessed in any order. Users are not required to complete one module before proceeding to the next. This flexible approach was designed to accommodate individual preferences and needs, allowing users to engage with the content that is most relevant to them at any given time. Other features of the app include a daily symptom tracker, data-driven health analytics, and on-demand lessons for a variety of topics such as managing the impact of menopause on work and personal relationships. Personalized health tips and recommendations are provided to participants based on their individual symptom logs. Participants can opt in to daily reminders and notifications to encourage regular engagement with the program.

### Statistical Analysis

#### Power Analysis

The sample size was determined based on a previous study examining hot flash severity as the primary outcome in a self-guided CBT intervention for menopausal symptoms in a similar general (not treatment-induced menopause) population [26]. G*power was used to calculate a sample size for a 3:1 allocation ratio with an effect size of 0.75, resulting in a sample size of 126 (94:32), therefore, a sample of 145 after accounting for 15% attrition would be adequately powered to test hypotheses [55].

#### Treatment Outcomes

Average hot flash severity (HFRS total score) served as the primary outcome measure and was submitted to a repeated measures ANOVA where time (T1, T2, and T3) was the within-subjects factor and treatment arm (Caria, control) was the between-subjects factor.

Exploratory outcomes were examined by subgroups (ie, participants with cutoffs reflective of depression, anxiety, or poor sleep quality in the clinical range). This was done to examine the intervention impact on those with symptom levels indicative of a need for treatment. Since analyses based on subgroups reduced the power to detect differences between treatment arms, a planned comparison approach was used. Specifically, repeated measures ANOVA, where time (T1, T2, and T3) was the within-subjects factor, was performed within each treatment arm. Significant main effects of time were followed up with 2-tailed paired *t* tests to aid in the interpretation of results. We also evaluated whether participants who completed all 6 weeks of treatment significantly differed from noncompleters in terms of clinical characteristics (eg, hot flush severity). We did not expect differences; however, it is important to evaluate to determine whether premature discontinuation was driven by differences in clinical severity.

All analysis was 2-tailed with the α level set to .05. Participants with incomplete data were omitted from the analysis. Statistical parameters include central tendency (ie, means) and SD for variance. Significant omnibus findings (eg, ANOVA) were followed-up with simple effects analysis (ie, 2-tailed independent *t* tests and paired *t* tests). For the primary outcome measure, hot flash severity, Bonferroni was used to adjust for multiple comparisons. Hedges *g* (mean baseline minus mean posttreatment or SD pooled) was used as the effect size for primary and exploratory outcome measures. Analysis was performed in SPSS (version 27; IBM Corp).

## Results

### Overview

**Figure 2.**
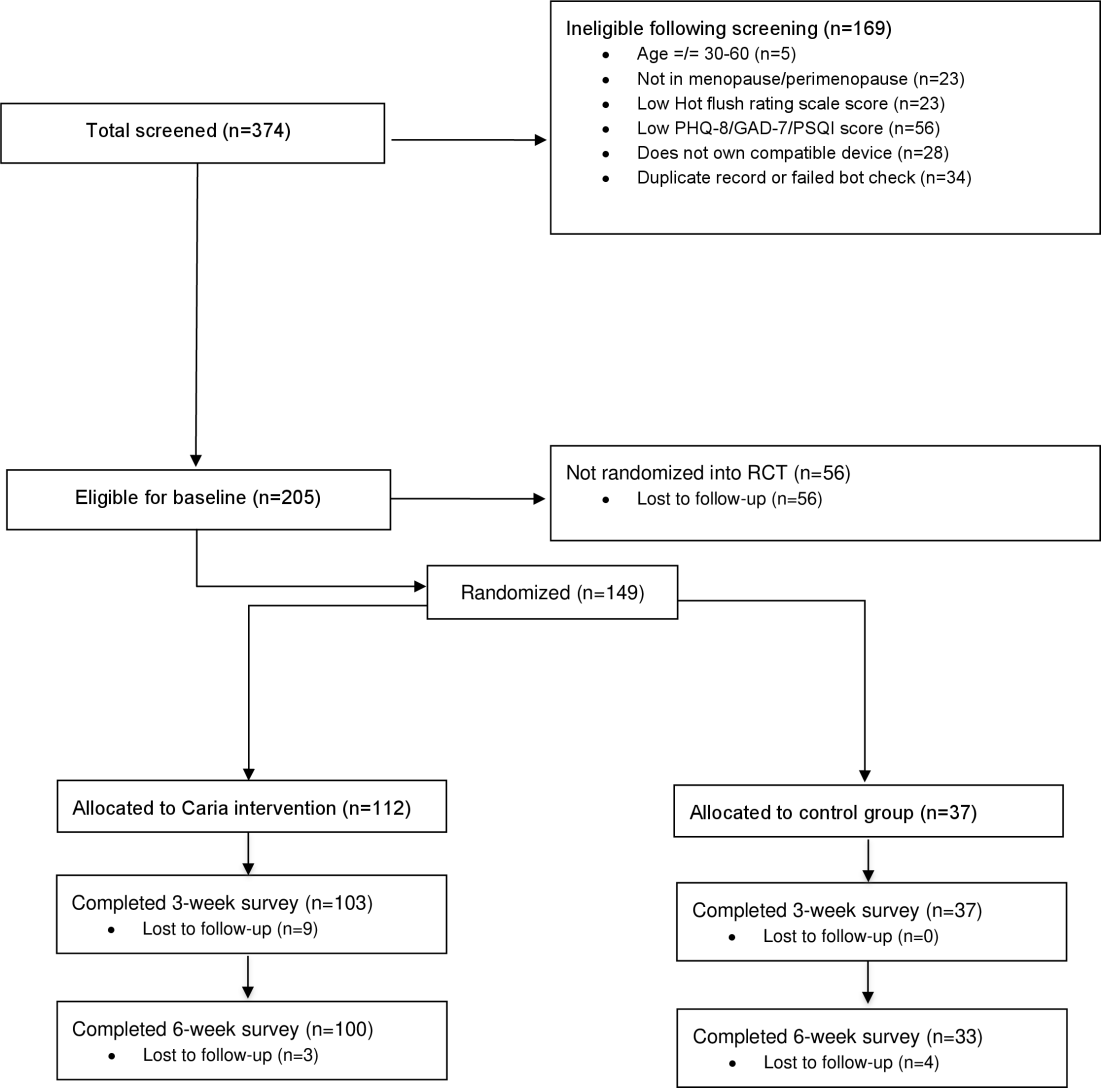
Study flowchart according to CONSORT. CONSORT: Consolidated Standards of Reporting Trials; GAD-7: Generalized Anxiety Disorder-7; PHQ-8: Patient Health Questionnaire Depression Scale-8; PSQI: Pittsburgh Sleep Quality Index; RCT: randomized controlled trial.

Eligibility screening was conducted for 374 participants. A total of 169 participants were not eligible to participate, and 56 participants were lost to follow-up. In total, 149 participants were randomly assigned to either the active DCP (n=112) or the control group (n=37). Figure 2 shows the consort diagram with details of the group allocation and follow-up.

### User Statistics

Participants were recruited from the United States and were between the ages of 30 and 60 (mean 47.68, SD 7.69) years. Among the sample, 86 (57.7%) participants were in perimenopause (as defined by having a period in the last 12 mo or irregular periods) and the remaining 63 (42.3%) were postmenopausal. The racial breakdown of the participants was as follows: 118 (79.2%) White, 13 (8.8%) Black/African American, 9 (6%) Mixed, 7 (4.7%) Asian, and 2 (1.3%) American Indian/Alaskan Native, out of a total of 149 participants. Racial classification terms were provided by researchers, and responses were selected by participants. Table 1 shows the participant demographics.

### Baseline Characteristics

Participants randomized to the active Caria arm (n=112) had marginally lower hot flash severity than those assigned to the control arm (n=37; *t*_147_=1.97; *P*=.05); frequency of hot flashes did not differ (*t*_147_=1.2; *P*=.23) nor did depression, anxiety, or sleep quality (lowest *P*=.34) between treatment conditions. Regarding demographic characteristics, participants in the Caria versus control arm did not differ in age (*t*_147_=.67; *P*=.97), level of education in years (*t*_147_=.11; *P*=.91), or race (*χ*^2^_4_=3.3; *P*=.51).

When evaluating clinical depression (ie, participants with PHQ-8 total score ≥10), average depression did not differ between participants in the Caria condition (n=56 mean 14.4, SD 3.9) and control condition (n=21; mean 15.0, SD 3.7; *t*_75_=0.6; *P*=.50). For clinical anxiety (ie, participants with GAD-7 total score ≥10), average anxiety did not differ between participants in the Caria condition (n=39; mean 14.9, SD 3.2) and control condition (n=15; mean 16.0, SD =3.8; *t*_52_=1.1; *P*=.29). Concerning clinical poor sleep quality (ie, participants with PSQI global score ≥5), average sleep quality did not differ between participants in the Caria condition (n=102; mean 11.0, SD 2.9) and control condition (n=35; mean 11.5, SD 3.7*; t*_135_=0.9; *P*=.37).

### Engagement

Engagement with the DCP was tracked by measuring the number of unique sessions and time spent on the app. Over the course of the 6-week intervention, participants logged into the Caria app an average of 53.2 times and spent 3.8 hours on the app. On average, the time between the first and last login was 30 days. Data were not collected on access to the control educational materials. Table 2 provides more information.

### Treatment Outcome

Participants who completed all 6 weeks of treatment (133/149, 89%) were similar to noncompleters (16/149, 10.7%) in terms of baseline hot flash severity (*t*_147_=1.18; *P*=.23), depression (*t*_146_=.38; *P*=.69), anxiety (*t*_147_=.04; *P*=.96), and sleep quality (*t*_147_=1.45; *P*=.14). Regarding hot flash frequency, baseline frequency was marginally lower for completers relative to noncompleters (*t*_147_=1.9; *P*=.05). Finally, there was no difference with regard to distribution of completers or noncompleters between treatment conditions (*χ*^2^_1_=0.0; *P*=.98).

### Primary Outcome Measure

Repeated measures ANOVA results for evaluating change in hot flash severity revealed main effects for time (*F*_2,262_=9.82; *P*<.001) and treatment arm (*F*_1,131_=6.08; *P*=.01), and there was a significant time × treatment arm interaction (*F*_2,262_=3.23; *P*=.04). To evaluate the main effect of time, treatment arm was collapsed and 2-tailed paired *t* tests showed hot flash severity significantly decreased from baseline to week 3 (*t*_139_=4.67; *P*<.001) but not from weeks 3 to 6 (*t*_132_=1.25; *P*=.21). The significant finding was significant when adjusting for multiple comparisons (*P*=.02). To examine the main effect of the treatment arm, time was collapsed, and 2-tailed independent *t* tests revealed participants in the Caria treatment arm had less hot flash severity than participants in the control arm (*t*_147_=2.72; *P*=.007). The significant finding was significant when adjusting for multiple comparisons (*P*=.02).

To explore the interaction, 2-tailed paired *t* tests were performed within each treatment arm. Participants in the Caria arm showed a significant decrease in hot flash severity from baseline to week 3 (*t*_102_=3.54; *P*=.001) and from weeks 3 to 6 (*t*_99_=2.90; *P*=.005). Findings were significant when adjusting for multiple comparisons (*P*=.02). Results for the control arm revealed a significant decrease in hot flash severity from baseline to week 3 (*t*_36_=3.33; *P*=.002) but not from weeks 3 to 6 (*t*_32_=1.19; *P*=.24). The significant finding was significant when adjusting for multiple comparisons (*P*=.02). Two-tailed independent *t* test analysis for each time point showed the marginally lower baseline hot flash severity for participants eventually assigned to the Caria relative to control arm reported previously (*t*_147_=1.97; *P*=.05) and no significant treatment arm effects were observed at week 3 (*t*_138_=1.70; *P*=.09). However, after completing treatment (ie, week 6), participants in the active treatment arm exhibited less hot flash severity than participants in the control arm (*t*_131_=3.21; *P*=.002). The significant week 6 finding was significant when adjusting for multiple comparisons (*P*=.02).

In summary, participants randomized to the Caria treatment arm exhibited significantly greater improvement in hot flashes after completing 6 weeks of treatment relative to participants randomized to the control arm.

### Exploratory Outcome Measures

#### Depression

For participants with a PHQ-8 total score ≥10 (77/149, 51.6%), the repeated measures ANOVA of change in PHQ-8 scores showed a main effect of time for participants in the Caria treatment arm (*F*_2,96_=15.2; *P*<.001) but not control arm (*F*_2,40_=2.0; *P*=.15). Follow-up paired *t* tests for participants in the Caria treatment arm showed depression decreased from baseline to week 3 (*t*_49_=3.3; *P*=.002). Depression also decreased from weeks 3 to 6 (*t*_48_=2.3; *P*=.02).

#### Sleep Quality

For participants with a PSQI global score ≥5 (137/149, 91.9%), the repeated measures ANOVA of PSQI scores showed a main effect of time for participants in the Caria treatment arm (*F*_2,186_=7.8; *P*=.001) but not control arm (*F*_2,62_=1.3; *P*=.28). Follow-up paired *t* tests for the Caria condition showed poor sleep quality significantly decreased from baseline to week 3 (*t*_95_=3.9; *P*<.001). Yet, there was no change in sleep quality from weeks 3 to 6 (*t*_93_=0.2; *P*=.81).

#### Anxiety

For participants with a GAD-7 total score ≥10 (54/149, 36.2%), the repeated measures ANOVA of change in GAD-7 scores showed a main effect of time for participants in the Caria treatment arm (*F*_2,68_=5.0; *P*=.01) and control arm (*F*_2,26_=3.8; *P*=.03). In the Caria treatment arm, follow-up paired *t* tests showed anxiety significantly decreased from baseline to week 3 (*t*_34_=2.4; *P*=.02). Yet, there was no change in anxiety from weeks 3 to 6 (*t*_34_=0.9; *P*=.36).

Results were similar in the control arm. Specifically, follow-up paired *t* tests showed anxiety significantly decreased from baseline to week 3 (*t*_14_=2.6; *P*=.02). However, there was no change in anxiety from weeks 3 to 6 (*t*_13_=1.6; *P*=.13).

In summary, in the clinically depressed cohort, depression decreased across all time points within the Caria condition but not the control condition. In contrast, for clinically anxious participants, anxiety decreased during the early, but not later, phase of treatment, regardless of treatment arm. Finally, for participants with poor clinical sleep quality, sleep improved within the active condition but not the control condition. In addition, the significant improvement in the active condition was driven by the change in the early phase of treatment. Table 3 provides the details for all measures at all time points for each treatment arm.

**Table 3.** Group means, SDs, and *P* values comparing timepoints with effect sizes of participants randomized to use the DCP^a^ versus a control.

Outcome	Group					
	Baseline, mean (SD)	3 weeks, mean (SD)	*P* value (comparing baseline to 3 weeks)	6 weeks, mean (SD)	*P* value (comparing 3 weeks to 6 weeks)	Effect Size (Hedges *g*; baseline to 6 weeks)
Primary outcomes						
HFRS^b^—Problem						0.64
Caria DCP (n=103)	16.4 (6.7)	14.7 (6.3)^c^	.001	13.6 (6.6)^c^	.005	
Control (n=37)	19.1 (7.3)	16.9 (7.4)^c^	.002	17.8 (7.2)	.24	
HFRS—Frequency						0.02
Caria DCP (n=103)	2.8 (2.7)	2.5 (4.1)	.50	2.9 (5.4)	.61	
Control (n=37)	2.3 (3.2)	2.2 (2.7)	.94	2.7 (2.7)	.30	
Exploratory outcomes						
Depression (PHQ-8)						0.45
Caria DCP (n=50)^d^	14.0 (3.8)	12.2 (4.7)^c^	.002	11.2(5.3)^c^	.02	
Control (n=21)	15.0 (3.7)	13.3 (4.5)	.12	13.4 (4.1)	.93	
Sleep Disturbance (PSQI)						0.25
Caria DCP (n=100)^e^	10.7 (3.1)	10.0 (3.4)^c^	<.001	10.0 (3.5)	.81	
Control (n=35)^f^	11.5 (3.7)	10.9 (4.0)	.10	11.0 (3.7)	.82	
Anxiety (GAD-7)						0.33
Caria DCP (n=35)	14.8 (3.3)	13.3 (4.7)^c^	.02	12.7 (5.5)	.36	
Control (n=15)^g^	16.0 (3.8)	12.9 (5.4)^c^	.02	14.5 (4.5)	.13	

a DCP: digital care program.

b HFRS: Hot Flush Rating Scale.

c *P*<.05

d For 3 and 6 weeks, n=49.

e For 3 and 6 weeks, n=97.

f For 3 and 6 weeks, n=32.

g For 3 and 6 weeks, n=14.

## Discussion

### Principal Results

This study is the first evaluation of a novel smartphone-delivered DCP, Caria, in women with symptoms of menopause. Results from this RCT supported the primary hypothesis as distress related to hot flashes significantly decreased in participants randomized to the Caria arm compared to the control arm. Exploratory clinical measures of depression and sleep problems were also shown to improve in participants in the DCP arm but not in the control arm. The depression effect size was comparable to therapist-delivered interventions for depression in similar mildly distressed populations [56,57], suggesting that the digital behavioral intervention that uses CBT and mindfulness strategies may be an effective treatment for individuals experiencing symptoms related to perimenopause or menopause. This supports a digital, self-guided model of menopause-related care.

Specifically, symptoms of distress from hot flashes and depression demonstrated improvement in the DCP group over 6 weeks but not in the control group. Sleep disturbance for those in the DCP group improved over the first 3 weeks and remained stable through treatment, while there was no change in the control group. For those who used the DCP, distress from hot flashes decreased by 17.1%, depression symptoms decreased by 12.3%, anxiety symptoms decreased by 14.3%, and sleep problems decreased by 5.9%. Those with elevated anxiety symptoms who used Caria also showed a decrease in symptoms, but so did those in the control group. While there was a change in anxiety symptoms and a small to moderate effect size (*g*=0.33) for DCP use, the small sample size of those with elevated symptoms makes drawing meaningful conclusions difficult.

Clinical measures were examined from baseline to 3 weeks (midpoint) and 3 weeks to 6 weeks (posttreatment) in order to explore the length of treatment needed and change over time. In this sample, participants in the control group did experience improvement in hot flash symptoms and anxiety in the first 3 weeks of the study, suggesting there may have been value in the web-based educational material related to menopause symptoms (the control intervention), as it validates the symptoms experienced. It could also be a placebo response to participation in a research trial. However, those who used Caria showed additional improvement in hot flash distress and depression in the second half of the intervention (3-6 wk), while there was no change among participants in the control group. In addition, a significant improvement in sleep among those in the Caria group occurred during the first 3 weeks of intervention, while no change occurred in the control group. Overall, findings support that the DCP offers valuable intervention beyond web-based educational material and that 6 weeks of intervention provides more benefit than 3 weeks.

Notably, the frequency of hot flashes did not change significantly in this study; however, there was a reduction in the distress associated with these symptoms. This is a significant finding, given that the level of perceived distress caused by VMS is linked to quality of life and the likelihood of seeking health care services, rather than the frequency [58]. This study demonstrates that the DCP, despite not directly impacting the number of hot flashes experienced, effectively alleviates distress and consequently offers a promising avenue for enhancing overall quality of life and impacting health care utilization. It provides an option for women who prefer nonpharmacological interventions [14].

With regard to depression, depressive symptoms typically increase throughout the menopause transition, and depression rates are higher in women with VMS [59]. Clinical guidelines recommend evidence-based psychotherapies such as CBT as the primary treatment for depressive symptoms during the menopause transition [28]. However, long waitlists, high costs of care, and difficulty finding mental health providers with expertise in menopause limit women’s ability to receive adequate treatment. Given that this is a high-risk period in a woman’s life, a low-intensity intervention that can be easily disseminated has the potential for significant impact.

The effect size of Caria for depressive symptoms (effect size, *g*=0.45) was comparable to therapist-delivered interventions for a similar level of mild depressive symptoms (effect size, *d*=0.42) and better than many unguided digital interventions for depression (effect size, *g*=0.27) [56,57]. Intervening when depressive symptoms are mild may prevent the development of more severe episodes, yielding substantial benefits for patients, families, and the health care system. Individuals with mild depressive symptoms have been found to derive benefit from unguided interventions [60], and our data further supports the value of a self-guided DCP for menopause-related behavioral health that can be easily accessed.

As for sleep, approximately 80% of women who experience hot flashes have difficulty sleeping, and more severe hot flashes make it difficult to maintain sleep [61]. For those in the Caria group, improvement in sleep occurred over the first 3 weeks of treatment, and then, improvements remained stable during the second half of the intervention. The premise of CBT is that changing how one perceives an experience may change how one feels or behaves in response. Thus, it is possible that decreased distress about hot flashes (ie, improved coping related to hot flashes) demonstrated by Caria participants may lead to improved sleep through a better ability to respond to the experience of hot flashes. However, further research is needed to test this proposed mechanism of action.

It is also important to note that comorbidities and concurrent symptoms are a uniquely challenging aspect of menopause for women in midlife, with a significant impact on women’s well-being and productivity. It is the presence of concurrent menopausal symptoms that has been linked to productivity losses in the workplace, rather than a specific symptom [5]. In this sample, all participants had elevated levels of perceived distress from hot flashes, as well as one additional elevated mental health symptom. However, the majority of our participants had considerable levels of comorbidity; 33% (49/149) of participants had elevated levels of sleep difficulties, depression, and anxiety; 19% (28/149) had both elevated sleep difficulties and depression; and 3% (4/149) had both sleep difficulties and anxiety. The findings of this study and improvement in multiple symptoms point to the importance of digital interventions for menopause, taking a comprehensive approach to symptom management and targeting multiple concerns.

Sleep disturbances, in particular, have far-reaching effects as they have been associated with mood fluctuations, memory problems, obesity, and other cardiovascular risk factors, which are prevalent among women in midlife [62]. The noteworthy results of this study in demonstrating improvement in sleep-related symptoms hold significant promise, as addressing sleep issues may offer a potential pathway for enhancing overall health outcomes in menopausal women, including the management of other health conditions.

In addition to symptom improvement, participants in the DCP arm demonstrated consistent and high levels of engagement while reporting favorable levels of usability. Participants rated ease of use, ease of learning, usefulness, and satisfaction around 5 out of a 7-point scale (with 7 being most favorable). Participants engaged with the DCP for an average of 30 days in the 42-day trial, with a mean of 53.2 (SD 66.97) logins. Engagement and use of the Caria app are notable, particularly given the low rates of engagement typically found in health apps. A 2019 study examining mental health apps with similar components to Caria (mindfulness, peer support, and tracking) found 30-day retention rates of only 3.3% [63].

### Comparison With Prior Work

Although there are a few other self-guided digital interventions for menopause symptoms, the DCP performs favorably in comparison, with a higher effect for hot flash distress, and a similar impact on sleep [39]. The small to moderate range of symptom reduction in this study may be attributed to the relatively low baseline symptom severity reported by the majority of participants in the sample, resulting in less room for substantial improvement in symptom outcomes. While the effect sizes observed in the study were in the small to moderate range, it is worth noting the potential impact. By effectively addressing mild symptoms, self-guided DCPs like the one evaluated in this study have the potential to prevent symptom exacerbation into more severe and costly conditions and play a role in alleviating the burden on outpatient health services and associated health care costs [64-66].

Unlike many existing interventions that rely on evidence-informed content without rigorous testing, this study assessed the efficacy of the DCP using the gold-standard RCT methodology. Another significant strength of this study is the highly engaging design and self-guided delivery of the intervention. Participants demonstrated a high level of adherence and engagement with the intervention without the need for external support or coaching, suggesting the potential for high scalability. Furthermore, this study examined a range of symptoms that commonly impact women in midlife. Given the complexity of the menopausal transition, the comprehensive assessment of various aspects of physical and psychological health in this study provides a more holistic understanding of the intervention’s effectiveness in addressing the multifaceted challenges faced by women in midlife.

### Limitations

This trial has certain limitations that warrant consideration. First, participants in this study exhibited relatively low levels of behavioral health problems (anxiety, depression, and sleep disturbance) at baseline. Testing the DCP in a population with higher levels of symptoms would provide valuable insights into its effectiveness in addressing more severe symptoms and determining its suitability for a broader range of individuals. The predominantly White (118/149, 79%) and highly educated (89/149, 60% with college education or higher) sample may limit the generalizability of the findings. Future studies should aim to test the DCP in a more diverse sample to better capture the experiences of underrepresented populations. Recruitment exclusively through digital advertisements may have introduced a bias toward participants who are comfortable using technology and open to receiving health care in a digital format. As a result, the findings may not fully generalize to a broader population that may have varying levels of digital literacy or preferences for alternative modes of health care delivery. Additionally, the app’s infrastructure did not allow for monitoring the completion of specific modules, therefore, we were unable to measure adherence to individual components. Future research can benefit from more granular tracking features on the app. Neither participants nor researchers were blinded to the treatment condition. This can lead to overestimation of treatment effects. Finally, the use of self-report measures and the lack of long-term follow-up are limitations of the study. Future studies should incorporate posttreatment follow-up assessments with clinician-administered assessments to evaluate the sustained effects of the intervention over an extended period.

### Further Research

Future research can also build upon these findings and explore additional enhancements to the DCP, such as the incorporation of wearables and AI. Wearables like smartwatches or fitness trackers can monitor symptoms such as sleep disturbances, heart rate, and body temperature, and have the potential to provide objective and continuous data on vasomotor and behavioral health symptoms [67,68]. Combined with AI and software apps, they can also facilitate timely interventions and personalized recommendations. For instance, AI-powered systems can identify specific triggers or patterns that exacerbate symptoms and provide tailored strategies for managing them. This approach opens up new possibilities for targeted interventions and improving health outcomes.

### Conclusions

In conclusion, this study evaluated the efficacy of a 6-week DCP for common symptoms of menopause and found significant improvements in distress from VMS, depression, and sleep problems. The findings reinforce the significant impact of menopausal symptoms and validate the potential of digital interventions to address these challenges effectively. By leveraging the advantages of mobile technology and evidence-based interventions, the DCP provided a more scalable and accessible approach to support women in midlife experiencing symptoms of menopause. The improvements observed in this study contribute to the growing body of evidence supporting the effectiveness of digital behavioral interventions and fill a gap in the research on digital interventions targeted at women in midlife.

### Note on Terminology

This paper uses the term “women” in concordance with the World Health Organization. [69] However, it is important to acknowledge that people who identify as trans and nonbinary also experience menopause. There is currently a scarcity of readily available data on these populations. Further research efforts are required to better understand and address the unique experiences and challenges faced by nonbinary and transgender individuals in the context of menopause.

## Supplementary material

10.2196/58204 Checklist 1 CONSORT-EHEALTH checklist (V 1.6.1).

## Abbreviations

AI: artificial intelligence
CBT: cognitive behavioral therapy
CONSORT: Consolidated Standards of Reporting Trials
DCP: digital care program
GAD-7: Generalized Anxiety Disorder-7
HFRS: Hot Flush Rating Scale
PHQ-8: Patient Health Questionnaire Depression Scale-8
PSQI: Pittsburgh Sleep Quality Index
RCT: randomized controlled trial
USE: Usefulness, Satisfaction, and Ease Questionnaire
VMS: vasomotor symptoms
